# Irrigation with Magnetized Water Alleviates the Harmful Effect of Saline–Alkaline Stress on Rice Seedlings

**DOI:** 10.3390/ijms231710048

**Published:** 2022-09-02

**Authors:** Changkun Ma, Qian Li, Zhaoxin Song, Lijun Su, Wanghai Tao, Beibei Zhou, Quanjiu Wang

**Affiliations:** 1 State Key Laboratory of Eco-Hydraulics in Northwest Arid Region, Xi’an University of Technology, Xi’an 710048, China; 2 College of Horticulture, Northwest A&F University, Yangling, Xianyang 712100, China

**Keywords:** magnetized water, saline–alkaline stress, nitrogen absorption, rice (*Oryza sativa*)

## Abstract

Saline–alkaline stress suppresses rice growth and threatens crop production. Despite substantial research on rice’s tolerance to saline–alkaline stress, fewer studies have examined the impact of magnetic water treatments on saline–alkaline-stressed rice plants. We explored the physiological and molecular mechanisms involved in saline–alkaline stress tolerance enhancement via irrigation with magnetized water using Nipponbare. The growth of Nipponbare plants was inhibited by saline–alkaline stress, but this inhibition was alleviated by irrigating the plants with magnetized water, as evidenced by greater plant height, biomass, chlorophyll content, photosynthetic rates, and root system in plants irrigated with magnetized water compared to those irrigated with non-magnetized water. Plants that were irrigated with magnetized water were able to acquire more total nitrogen. In addition, we proved that rice seedlings irrigated with magnetized water had a greater root NO_3_^−^-nitrogen concentration and root NH_4_^+^-nitrogen concentration than plants irrigated with non-magnetized water. These findings suggest that treatment with magnetized water could increase nitrogen uptake. To test this hypothesis, we analyzed the expression levels of genes involved in nitrogen acquisition. The expression levels of *OsNRT1;1*, *OsNRT1;2*, *OsNRT2;1*, *OsAMT1;2*, *OsAMT2;1*, *OsAMT2;2*, *OsAMT2;3*, *OsAMT3;1*, *OsAMT3;2*, and *OsAMT3;3* were higher in plants exposed to magnetized water medium compared to those exposed to non-magnetized water media. We further demonstrated that treatment with magnetized water increases available nitrogen, NO_3_^−^-nitrogen content, and NH_4_^+^-nitrogen content in soil under saline–alkaline stress. Our results revealed that the increased resistance of rice seedlings to saline–alkaline stress may be attributable to a very effective nitrogen acquisition system enhanced by magnetized water.

## 1. Introduction

Soil saline–alkalization is abiotic stress that severely threatens crop production worldwide [1]. Approximately 20% of the world’s arable land suffers from saline–alkaline stress [2]. More than 90% of rice paddies suffer from saline–alkaline stress [2]. About 50% of the land will be lost from soil salinization by the year 2050 due to climate change and inappropriate cultivation practices [3]. Rice (*Oryza sativa* L.), as an important cereal, is saline–alkaline-sensitive [4]. Therefore, elucidating the mechanisms by which rice plants respond and adapt to saline–alkaline stress is important for selecting and breeding rice genotypes capable of growth in saline–alkaline soils.

Plants suffering from saline–alkaline stress have issues related to high concentrations of neutral salts and high soil pH (due to increased accumulation of alkaline salts, i.e., Na_2_CO_3_ and NaHCO_3_) [5]. High concentrations of neutral salts promote Na^+^ accumulation in plants, decreasing nutrient acquisition and resulting in nutritional imbalances and oxidative damage. [1]. Consequently, plants have developed many physiological and biochemical mechanisms to respond and adapt to saline–alkaline stress. For instance, it has been reported that maintaining cellular ion homeostasis, osmotic homeostasis, reactive oxygen species (ROS) scavenging, organic acid accumulation, and H^+^ secretion are essential mechanisms that provide saline–alkaline stress tolerance in plants. [5,6,7,8]. However, relatively little attention has been paid in these investigations to the mechanisms underlying the effects of elevated soil pH on plant growth. While, numerous studies have conclusively demonstrated that the inhibitory effects of salinity–alkalinity stress on plant growth are more severe than those of neutral salt stress, primarily due to the higher pH value [9,10]. A high pH severely disrupts cell pH stability, destroys cell membrane integrity, and diminishes root vitality and photosynthetic function [11]. Furthermore, a high pH decreases the availability of minerals and organic matter in the soil, such as trace elements and plant-available nitrogen [12], causing nutritional stress in plants. Therefore, the combination of saline and alkaline stresses (particularly high pH) can result in a more severe trophic ion imbalance and inhibition of plant growth [9,13]. For example, under saline–alkaline stress, Bermuda grass slows nitrogen metabolism to maintain basic growth but at a slower growth rate [14]; chickpea (*Cicer arietinum* L.) increases its nodular mass to partially compensate for the inhibition of nitrogen absorption and metabolism [15]. In addition to those negative effects mentioned above, a high pH under saline–alkaline stress conditions will promote the conversion of NH_4_^+^ to NH_3_ in soil, resulting in the volatilization of ammonia and the loss of nitrogen from the soil [16]. Additionally, a high pH will decrease the potential nitrification rate and NO_3_^−^-nitrogen content of the soil [17]. As a result of these processes, the effective nitrogen available for plant absorption and use in the soil is drastically reduced, which has devastating effects on plant growth and development. [10]. In order to better endure low nitrogen conditions induced by saline–alkaline stress, plants have developed a variety of mechanisms over the course of evolution, for example, the higher nitrogen absorption mechanism by wheat under saline–alkaline stress conditions [18]. Through a hydroponic experiment, Zhang et al. (2020) investigated the impact of saline–alkaline stress on wheat nitrogen metabolism and discovered that higher nitrogen absorption of wheat is involved in tolerance of saline–alkaline stress, as alkaline stress-tolerance wheat genotypes are equipped with a higher nitrogen absorption capacity than saline–alkaline-non-tolerance wheat genotypes. In addition, several investigations have reported that iron acquisition and calcium metabolism all play key roles in saline–alkaline stress tolerance [5,6].

Nitrogen is an essential element for plant growth and development. In natural aerobic soil, nitrogen is usually taken up by land plants in the form of nitrate (NO_3_^−^) due to the significant nitrification of organic and fertilizer nitrogen [19,20]. In contrast to land plants, rice contains an abundance of aerenchyma cells in its roots that can transport oxygen from shoots to roots and aid in the bacterial conversion of ammonium to nitrate (nitrification) at the root surface and rhizosphere [20]. Therefore, ammonium and nitrate are two important sources of nitrogen for rice growth, and rice has developed two different mechanisms for absorbing nitrogen, one for ammonium and one for nitrate [20,21]. By modulating the expression and function of genes underlying these two nitrogen uptake systems, rice plants can modify their capacity to acquire different forms of nitrogen at the molecular level [20]. For NO_3_^−^ absorption, it was discovered that the *OsNRT1* and *OsNAR2/OsNRT2* families encode transporters responsible for the uptake of nitrates in higher plants [19,20]. According to the results of a recent gene sequencing study, 93 *OsNRT1*, 4 *OsNRT2,* and 2 *OsNAR2* genes were identified in rice plants [11]. And in contrast to OsNRT1, most OsNRT2 members need to connect to OsNAR2 to form a protein complex that can either transport or absorb nitrate [19]. While for NH_4_^+^ absorption, it was discovered that the AMT protein family was responsible for the absorption of NH_4_^+^ in the roots of rice plants, and at least ten members of the AMT family have been identified [20].

Magnetic water irrigation, as a promising and eco-friendly new technique, is now widely employed in the field of agriculture [22,23]. Previous studies have reported that irrigation with magnetized water can increase crop tolerance to stresses, such as salt stress and drought stress [24,25,26]. For instance, Zhang et al. (2022) found that magnetic water treatment reduces the saline stress of brackish water at the onset of cotton seedling development [27]. Selim et al. (2019) demonstrated that the application of magnetic water increased wheat growth and cereal yield under drought stress [28]. It was also reported that the promotion of plant growth under magnetized water treatments may be attributable to the fact that magnetized water facilitates the absorption of nutrients by plants [29]. As Selim et al. (2019) reported, irrigation with magnetized water under drought stress increased the nitrogen concentration of two wheat plant cultivars (*Triticum aestivum* L. cvs. Sakha 93 and Sids 9) by 28.6% and 19.0%, respectively. Teixeira da Silva (2014) concluded that irrigation with magnetized water improves the growth and development of plants quantitatively and qualitatively, which could be attributed to the increased efficiency of nutrient absorption by plants [30]. Effective nutrient absorption mechanisms that equip plants with abiotic stress tolerance may be the result of increasing the available nitrogen, phosphorus, and potassium to plants in stressed soils, as magnetized water irrigation enhanced fertilizer dissolution and deeper soil penetration [22]. In addition, plants irrigated with magnetized water have a larger capacity to tolerate salt and drought stress by generating more phytohormone, soluble sugar, free amino proline, and acids compared with plants irrigated with untreated water [26,28,29,31,32]. Recently, studies have been carried out to investigate the molecular and physiological mechanisms underlying the effect of magnetic water treatments on the development of plants and the germination of seeds when the plants are subjected to salt and drought stress. However, to the best of the authors’ knowledge, the effects of magnetized water on plant growth and seed germination under saline–alkaline stress have rarely been attempted. Therefore, in the present study, we analyzed the effects of magnetized water on the growth of Nipponbare seedlings in the condition of saline–alkaline stress and examined the mechanisms underlying the tolerance of rice plants to saline–alkaline stress enhanced by magnetized water treatment via the detection of nitrogen concentration and expression of genes involved in nitrogen uptake and root system architecture.

## 2. Results

### 2.1. Effects of Magnetized Water on Growth

**Figure 1:**
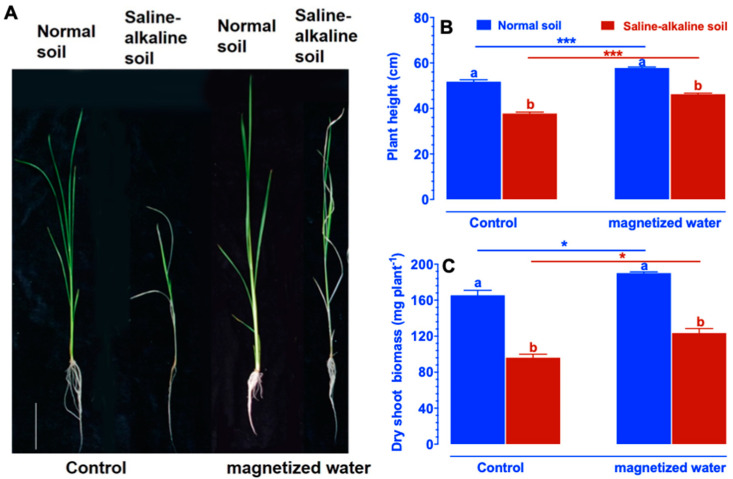
Effects of magnetized water on Nipponbare seedlings under saline–alkaline stress conditions. (**A**) Growth performance. (**B**) Plant height. (**C**) Dry shoot biomass. Two-week-old rice seedlings grown in standard culture solution were transplanted into soil containing 60 mM NaHCO_3_ and a pH of 8.5. Then, for two weeks, they were irrigated with magnetic water. Irrigation water was magnetized by flowing through a 3000 gauss magnetron device with a 0.5-inch diameter magnetic field. Bars = 10 cm. Daare means ± SE (*n* ≥ 4). Means with different letters are significantly different (*p <* 0.05) within the same treatment. Asterisks indicate significant differences between control and saline–alkaline stress of the same genotype, as determined by Student’s *t*-test (* *p <* 0.05, *** *p <* 0.001).

To investigate the effects of magnetized water on the growth of Nipponbare seedlings under saline–alkaline stress conditions, two-week-old rice seedlings were grown in soil enriched with 60 mM NaHCO_3_ and at a pH of 8.5 for two weeks. As shown in Figure 1A, saline–alkaline stress decreased the shoot and root growth of Nipponbare, but treatment with magnetized water ameliorated this impact. Saline–alkaline stress significantly decreased plant height and dry shoot biomass, whereas irrigation with magnetized water alleviated the decrease in plant height and dry shoot biomass (Figure 1B,C).

**Figure 2:**
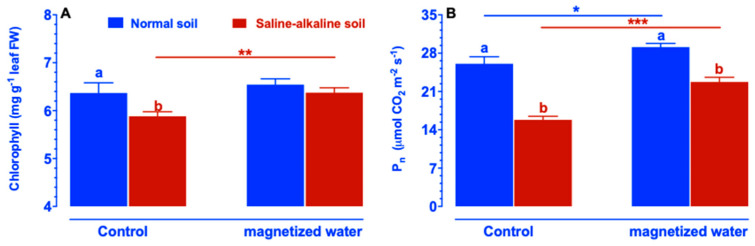
Foliar chlorophyll concentration (**A**) and photosynthetic rates (**B**) of Nipponbare plants cultivated in normal and saline–alkaline stress soil irrigated with magnetic and non-magnetic water. Treatments were as described in Figure 1. Means with different letters are significantly different (*p <* 0.05) within the same treatment. Asterisks indicate significant differences between control and saline–alkaline stress of the same genotype, as determined by Student’s *t*-test (* *p <* 0.05, ** *p <* 0.01, *** *p <* 0.001).

To determine the physiological mechanism by which plants treated with magnetized water grew better than plants treated with non-magnetized water, we examined the effects of magnetized water on foliar chlorophyll content and photosynthetic rates (Pn). Under non-magnetized water irrigation, the chlorophyll content and Pn of normal soil plants were much greater than those of saline–alkaline-stressed soil plants (Figure 2). Treatment with magnetized water increased chlorophyll concentration in plants grown in saline–alkaline soil, although there was no significant difference in chlorophyll concentration between plants grown in normal soil and those grown in saline–alkaline soil (Figure 2A). Following irrigation with magnetized water, the Pn of rice plants in both normal and saline–alkaline stress soils increased significantly (Figure 2B). These results suggest that irrigating rice plants with magnetized water can boost their growth under saline–alkaline stress.

### 2.2. Effects of Magnetized Water on Total Nitrogen, NO_3_^−^-Nitrogen, and NH_4_^+^-Nitrogen Concentration

**Figure 3:**
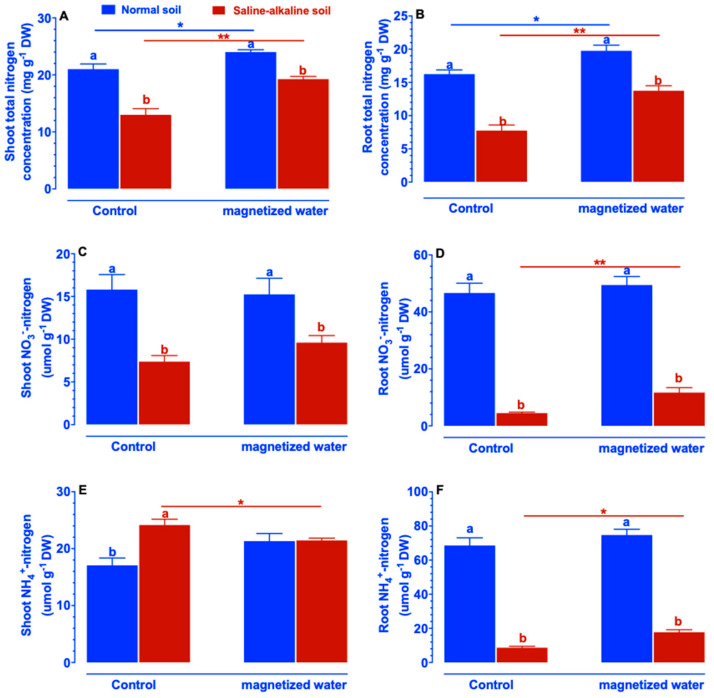
Effects of magnetized water on the concentration of total nitrogen (**A**,**B**), NO_3_^−^-nitrogen (**C**,**D**), and NH_4_^+^-nitrogen (**E**,**F**) in the shoots and roots of Nipponbare plants grown in normal and saline–alkaline stress soil. Treatments were as described in Figure 1. Means with different letters are significantly different (*p <* 0.05) within the same treatment. Asterisks indicate significant differences between control and saline–alkaline stress of the same genotype, as determined by Student’s *t*-test (* *p <* 0.05, ** *p <* 0.01).

Given that saline–alkaline-stressed rice plants have to cope with low nitrogen concentration in the growth substrate, the effects of saline–alkaline stress on the growth of rice seedlings will be alleviated by increased nitrogen absorption. We evaluated the effects of magnetized water on total nitrogen, NO_3_^−^-nitrogen, and NH_4_^+^-nitrogen concentration in shoots and roots of Nipponbare under both normal and saline–alkaline stress conditions. As demonstrated in Figure 3, saline–alkaline stress resulted in a considerable decrease in shoot/root total nitrogen and NO_3_^−^-nitrogen of Nipponbare seedlings irrigated with non-magnetized water, whereas with exposure to magnetized water, there was a significant increase in shoot/root total nitrogen of Nipponbare seedlings under both normal and saline–alkaline stress conditions (Figure 3A,B). In both normal and saline–alkaline stress conditions, the application of magnetized water had no effect on the NO_3_^−^-nitrogen content of Nipponbare shoots (Figure 3C). Irrigation with magnetized water led to a significant rise in root NO_3_^−^-nitrogen of Nipponbare under the condition of saline–alkaline stress, although there was no significant difference in normal soil when magnetized water was applied (Figure 3D). Under non-magnetized water conditions, the NH_4_^+^-nitrogen concentration in shoots of Nipponbare was increased by saline–alkaline stress, whereas the increase was mitigated via irrigation with magnetized water (Figure 3E). Under control conditions, the concentration of NH_4_^+^-nitrogen in the roots of Nipponbare plants growing in normal soil was higher than in saline–alkaline stress soil (Figure 3F). When irrigated with magnetized water, the root NH_4_^+^-nitrogen concentration of Nipponbare plants cultivated in saline–alkaline stress soil increased significantly, whereas the root NH_4_^+^-nitrogen concentration of Nipponbare plants planted in normal soil remained relatively steady (Figure 3F).

### 2.3. Effects of Magnetized Water on Root System Architecture

**Figure 4:**
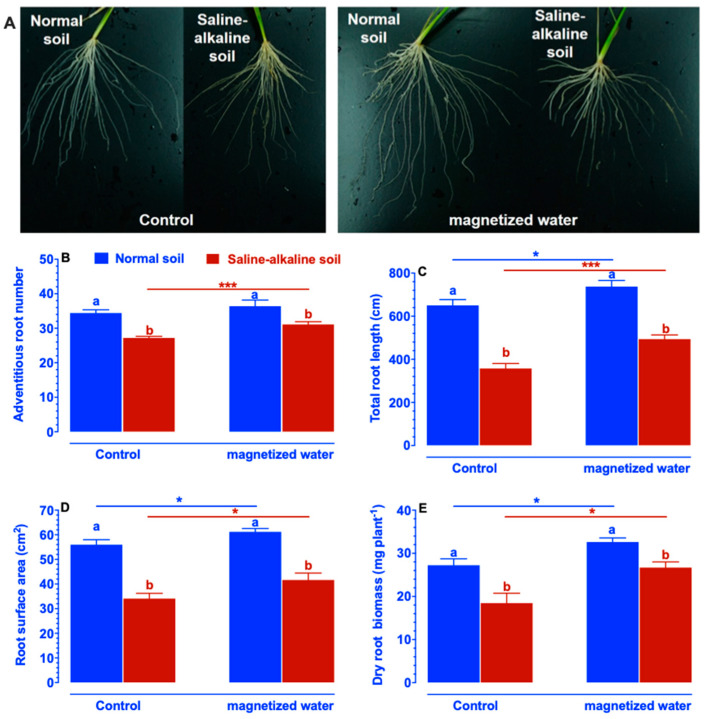
Effects of magnetized water on the root system architecture of Nipponbare plants grown in normal and saline–alkaline stress soil. (**A**) Root phenotypes. (**B**) Adventitious root number. (**C**) Total root length. (**D**) Root surface area. (**E**) Dry root biomass. Treatments were as described in Figure 1. Means with different letters are significantly different (*p <* 0.05) within the same treatment. Asterisks indicate significant differences between control and saline–alkaline stress of the same genotype, as determined by Student’s *t*-test (* *p <* 0.05, *** *p <* 0.001).

The root system architecture is an important trait responsible for efficient nitrogen acquisition. The fact that Nipponbare seedlings watered with magnetized water had a higher nitrogen concentration than those irrigated with non-magnetized water motivated us to examine if the root systems of Nipponbare seedlings responded differently to magnetized water irrigation. As shown in Figure 4, the adventitious root number, total root length, root surface area, and dry root biomass of Nipponbare plants cultivated in normal soil were greater than those of plants produced in saline–alkaline stress soil under control circumstances. The adventitious root number of Nipponbare plants cultivated in saline–alkaline stress soil increased significantly when irrigated with magnetized water, whereas the adventitious root number of Nipponbare plants grown in normal soil remained relatively steady when watered with magnetized water (Figure 4B). Irrigation with magnetized water led to a significant increase in total root length, root surface area, and dry root biomass of Nipponbare plants cultivated in normal and saline–alkaline stress soils (Figure 4C–E).

### 2.4. Effects of Magnetized Water on the Expression of Genes Involved in Nitrogen Uptake

**Figure 5:**
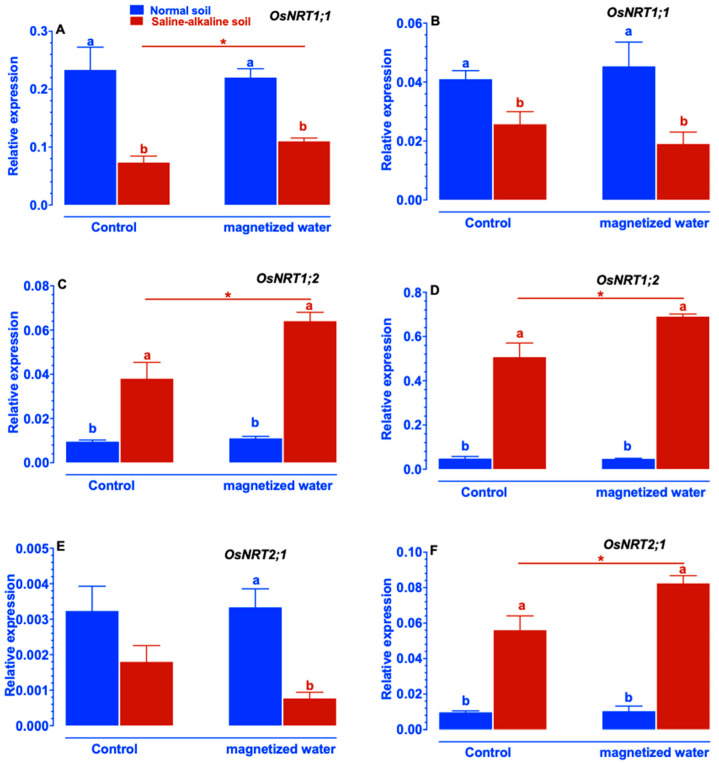
Effects of magnetized water on the expression of the *OsNRT* gene family in Nipponbare seedlings grown in normal and saline–alkaline stress soil: (**A**) *OsNRT1;1* in shoot, (**B**) *OsNRT1;1* in root, (**C**) *OsNRT1;2* in shoot, (**D**) *OsNRT1;2* in root, (**E**) *OsNRT1;3* in shoot, (**F**) *OsNRT1;3* in root. Treatments were as described in Figure 1. Means with different letters are significantly different (*p <* 0.05) within the same treatment. Asterisks indicate significant differences between control and saline–alkaline stress of the same genotype, as determined by Student’s *t*-test (* *p <* 0.05).

The increased ability of Nipponbare plants to acquire nitrogen in response to magnetized water shows that it may influence the expression of genes involved in nitrogen uptake. We identified alterations in the expression patterns of genes involved in nitrogen acquisition, such as the *OsNRT* and *OsATM* families encoding nitrite and ammonium transporters. Magnetized water significantly increased the expression levels of *OsNRT1;1* and *OsNRT1;2* in shoots of Nipponbare plants growing in saline–alkaline stress soil (Figure 5A,C), while the expression levels of *OsNRT2;1* in the shoots of Nipponbare plants were unaffected by exposure to magnetized water (Figure 5E). Magnetized water did not affect the expression levels of *OsNRT1;1* in the roots of Nipponbare plants (Figure 5B). Magnetized water greatly increased the expression levels of *OsNRT1;2* and *OsNRT2;1* in the roots of Nipponbare plants exposed to saline–alkaline soil stress (Figure 5D,F).

**Figure 6:**
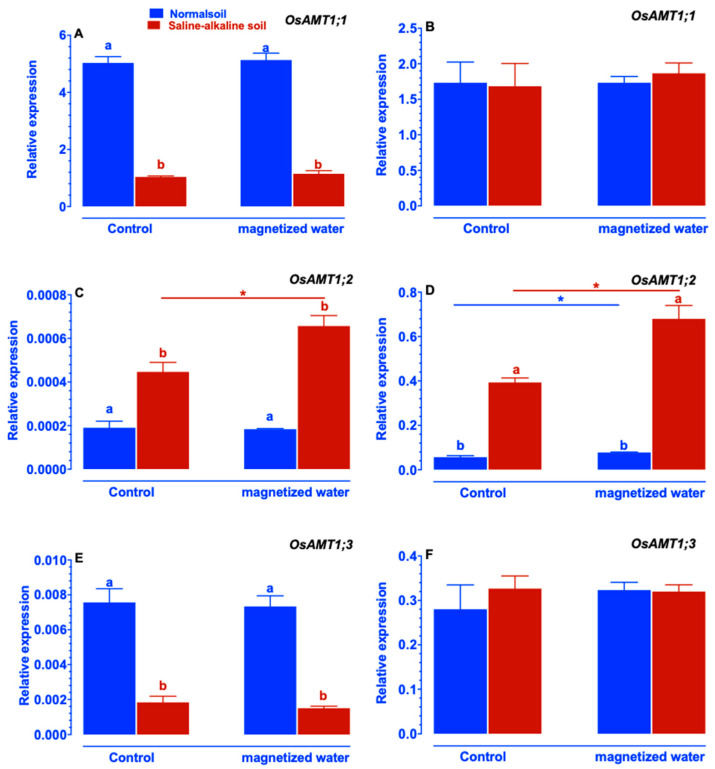
Effects of magnetized water on the expression of the *OsAMT1* gene family in Nipponbare seedlings grown in normal and saline–alkaline stress soil: (**A**) *OsAMT1;1* in shoot, (**B**) *OsAMT1;1* in root, (**C**) *OsAMT1;2* in shoot, (**D**) *OsAMT1;2* in root, (**E**) *OsAMT1;3* in shoot, (**F**) *OsAMT1;3* in root. Treatments were as described in Figure 1. Means with different letters are significantly different (*p <* 0.05) within the same treatment. Asterisks indicate significant differences between control and saline–alkaline stress of the same genotype, as determined by Student’s *t*-test (* *p <* 0.05).

Expression levels of *OsAMT1;1* and *OsAMT1;3* in Nipponbare plants were unaffected by exposure to magnetized water (Figure 6A,B,E,F). Magnetized water irrigation increased the expression of *OsAMT1;2* in both shoots and roots of saline–alkaline stressed Nipponbare plants (Figure 6C,D).

**Figure 7:**
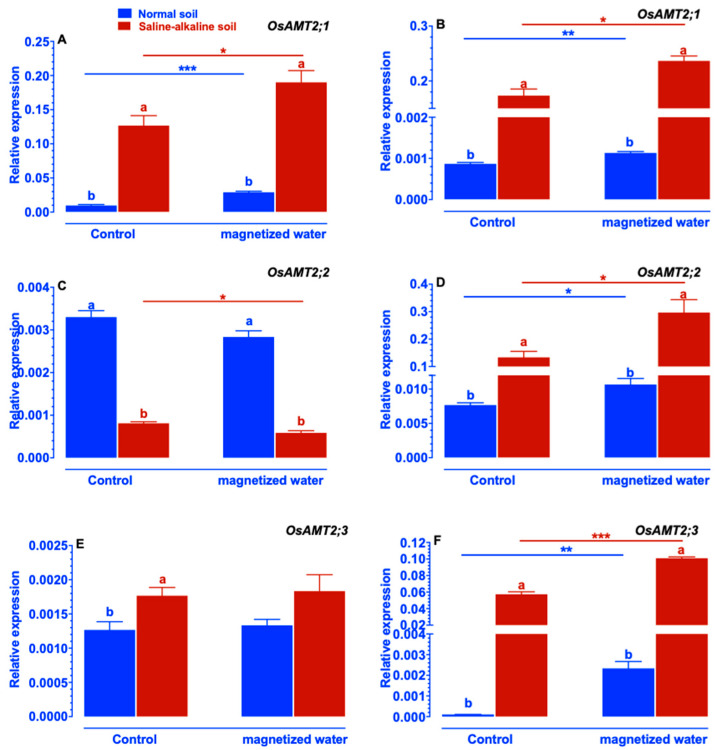
Effects of magnetized water on the expression of the *OsAMT2* gene family in Nipponbare seedlings grown in normal and saline–alkaline stress soil: (**A**) *OsAMT2;1* in shoot, (**B**) *OsAMT2;1* in root, (**C**) *OsAMT2;2* in shoot, (**D**) *OsAMT2;2* in root, (**E**) *OsAMT2;3* in shoot, (**F**) *OsAMT2;3* in root. Treatments were as described in Figure 1. Means with different letters are significantly different (*p <* 0.05) within the same treatment. Asterisks indicate significant differences between control and saline–alkaline stress of the same genotype, as determined by Student’s *t*-test (* *p <* 0.05, ** *p <* 0.01, *** *p <* 0.001).

Expression levels of *OsAMT2;1* and *OsAMT2;2* in shoots of Nipponbare plants growing in saline–alkaline stress soil were markedly upregulated by exposure to magnetized water (Figure 7A,C), while the expression levels of *OsAMT2;3* in shoots of Nipponbare plants were unaffected by exposure to magnetized water (Figure 7E). The expression of *OsAMT2;1*, *OsAMT2;2*, and *OsAMT2;3* in the roots of Nipponbare plants growing in both normal and saline–alkaline stress soil was increased by irrigation with magnetized water (Figure 7B,D,F).

**Figure 8:**
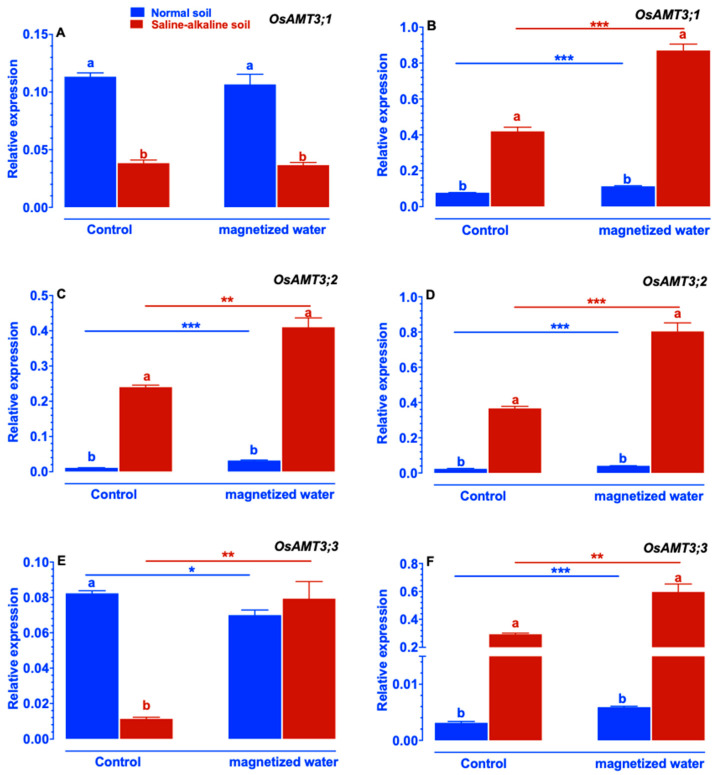
Effects of magnetized water on the expression of the *OsAMT3* gene family in Nipponbare seedlings grown in normal and saline–alkaline stress soil: (**A**) *OsAMT3;1* in shoot, (**B**) *OsAMT3;1* in root, (**C**) *OsAMT3;2* in shoot, (**D**) *OsAMT3;2* in root, (**E**) *OsAMT3;3* in shoot, (**F**) *OsAMT3;3* in root. Treatments were as described in Figure 1. Means with different letters are significantly different (*p <* 0.05) within the same treatment. Asterisks indicate significant differences between control and saline–alkaline stress of the same genotype, as determined by Student’s *t*-test (* *p <* 0.05, ** *p <* 0.01, *** *p <* 0.001).

Except for *OsAMT3;1* (Figure 8A), irrigation with magnetized water increased the expression of *OsAMT3;1*, *OsAMT3;2*, and *OsAMT3;3* in Nipponbare plants growing in both normal and saline–alkaline stress soil (Figure 8).

### 2.5. Effects of Magnetized Water on Total Nitrogen Content, Available Nitrogen Content, NO_3_^−^Nitrogen Content, and NH_4_^+^-Nitrogen Content

**Figure 9:**
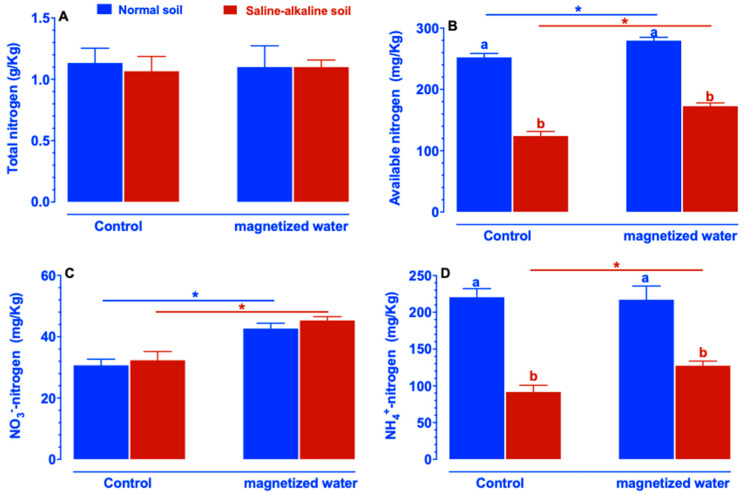
Effects of magnetized water on total nitrogen content (**A**), available nitrogen content (**B**), NO_3_^−^-nitrogen content (**C**), NH_4_^+^-nitrogen content (**D**) in soil. Soil samples were measured after treatment with magnetized water for five days. Means with different letters are significantly different (*p <* 0.05) within the same treatment. Asterisks indicate significant differences between control and saline–alkaline stress of the same genotype, as determined by Student’s *t*-test (* *p <* 0.05).

There are no variations in total nitrogen between normal and saline–alkaline stress soil after five days of treatment with magnetized water (Figure 9A). The content of available nitrogen in soil was decreased by saline–alkaline stress, but the decrease was alleviated by irrigation with magnetic water (Figure 9B). NO_3_*^−^*-nitrogen content in both normal and saline–alkaline stress soil was increased by exposing the soil to magnetic water (Figure 9C). NH_4_^+^-nitrogen content in normal soil was unaffected by magnetized water, whereas NH_4_^+^-nitrogen content in saline–alkaline stress soil increased after treatment with magnetized water (Figure 9D).

## 3. Discussion

Despite the worldwide prevalence of soil saline–alkalization, the physiological and molecular mechanisms by which plants respond to and adapt to saline–alkaline stress remain largely elusive. In particular, few investigations have focused on the impact of magnetic water treatments on plant growth under saline–alkaline stress. In this study, we investigate whether rice’s tolerance to saline–alkaline stress varied in response to magnetized water. The rice genotype Nipponbare was utilized in this study. We compared the effects of irrigation with magnetized water on Nipponbare seedlings grown in saline–alkaline stress soil to those grown in normal soil. Our results revealed that magnetized water enhanced the tolerance of Nipponbare seedlings to saline–alkaline stress, as evidenced by less plant height and shoot biomass loss in response to saline–alkaline stress (Figure 1). The enhancement of rice growth may account by the higher chlorophyll concentration and photosynthetic rates induced by irrigation with magnetized water (Figure 2). Effective nitrogen uptake and a larger root system induced by irrigation with magnetized water may also contribute to the increased growth of rice seedlings grown in a saline–alkaline environment. Previous studies have demonstrated that magnetized water irrigation not only alters the physicochemical properties of soil (such as lowering soil salinity and pH and increasing the availability of nitrogen and phosphorus [22,30]) but also promotes the growth of plants’ root systems [33], thereby increasing the nitrogen and phosphorus absorption by plants and promoting plant growth under stressful conditions [22,28,34]. Inconsistent with these findings, our research revealed that rice root parameters were less affected by saline–alkaline stress when they were exposed to magnetized water (Figure 4) and that the reduction in available nitrogen in the soil induced by saline–alkaline stress was also mitigated (Figure 9). The larger root system of rice seedlings induced by irrigation with magnetized water may facilitate their nitrogen acquisition and increase nitrogen concentration (Figure 3), thereby conferring tolerance to nitrogen deficiency caused by saline–alkaline stress. Furthermore, the genes that are involved in rice’s nitrogen acquisition were upregulated by the magnetized water (Figure 5, Figure 6, Figure 7 and Figure 8), which may facilitate nitrogen acquisition and increase nitrogen concentration in plants. In summary, our findings indicate that treatment with magnetized water improved the rice genotypes’ tolerance to saline–alkaline stress by promoting efficient nitrogen uptake and the development of a larger root system.

Nitrogen is an essential element for plant growth and productivity. Nitrate and ammonium are two important nitrogen sources for plant growth [19]. Nitrate must be reduced and converted into nitrite by nitrate reductase in the cytoplasm of the cell, and nitrite must be reduced and converted into ammonium by nitrite reductase in plastids or chloroplasts that can be acquired by higher plants [19]. Previously, studies have reported that saline–alkaline stress interfered with nitrogen uptake by plants [7,18], and irrigation with magnetized water can mitigate the negative influence of saline–alkaline stress and increase the nitrogen uptake of plants [28]. However, neither the variation in nitrogen uptake by the roots and shoots nor the form of nitrogen (NO_3_^−^ or NH_4_^+^) absorbed by plants was examined in these investigations. In this study, we evaluated the impacts of magnetized water on the total nitrogen content of rice roots and shoots. Additionally, we compared the effects of magnetized water on particular nitrogen forms (NO_3_^−^ or NH_4_^+^) in rice roots and shoots. Our results indicated that irrigation with magnetized water increased total nitrogen concentration in both rice shoots and roots under saline–alkaline stress (Figure 3A,B). Our results also indicated that magnetized water treatment facilitated the uptake of NO_3_^−^ by rice under saline–alkaline stress conditions, as evidenced by a significant increase in NO_3_^−^ content in roots and a non-significant increase in NO_3_^−^ content in shoots (Figure 3C,D). This may potentially make rice more tolerant to saline–alkaline stress. Inconsistent with our results, previous studies also reported that absorption of NO_3_^−^ by plants plays an important role in the tolerance of saline–alkaline stress. For example, Fu et al. (2018) discovered that *Trichoderma asperellum* mitigated the negative effects of saline–alkaline stress on maize seedlings by increasing NO_3_^−^ levels in the leaves and roots [7]. Zhang et al. (2014) reported that the application of exogenous spermidine to tomato seedlings mitigated the degree of a decline in NO_3_^−^-nitrogen content in response to saline–alkaline stress [35]. To further investigate the molecular mechanisms underlying the increased NO_3_^−^-nitrogen content in roots of Nipponbare plants caused by the application of magnetized water, we examined the expression patterns of genes involved in NO_3_^−^ uptake. Generally, the *OsNRTs* gene families are responsible for NO_3_^−^ uptake by plant roots from the soil [19]. *OsNRT1* was the first cloned nitrate transporter in rice plants involved in nitrate uptake [36]. Subsequently, another ten *OsNRTs* genes have now been identified in rice. For instance, *OsNAR2;1*, *OsNRT2;1*, O*sNRT2;2*, and *OsNRT2;3* is involved in nitrate acquisition with low affinity in rice plants [20]. In most cases, the expression levels of *OsNRT2;1*, *OsNRT2;2*, and *OsNRT2;3* are up-regulated to facilitate nitrogen uptake by plants and increase the amount of nitrogen content in plant roots [16], which was also observed in our investigation. Gene expression analysis indicated that the expression levels of *OsNRT1;2* and *OsNRT2;1* in the root of rice plants were significantly up-regulated under a saline–alkaline stress environment, and the amount of the up-regulation was greater in plants when magnetized water was applied (Figure 5). Generally, saline–alkaline stress modifies the expression of *OsNRT1;1*, *OsNRT1;2*, and *OsNRT2;1* in plant shoots [37]. Inconsistent with these findings, our research also indicates that exposure to saline–alkaline stress modifies the expression of genes involved in NO_3_^−^ absorption in shoots of rice plants. This change could be the result of decreasing nitrogen levels in plants, with the up-regulation expression of these genes acting as compensation for the decrease in NO_3_^−^-nitrogen content in shoots. The higher expression levels of these genes induced by magnetized water may enable Nipponbare plants to acquire NO_3_^−^ more efficiently, hence contributing to their greater nitrogen accumulation under saline–alkaline stress.

It is widely recognized that an accumulation of NH_4_^+^ can cause ammonia toxicity in plants; therefore, reducing NH_4_^+^ concentration in plants can mitigate the damage caused by environmental stresses on plants [38]. Zhang et al. (2013) discovered that saline–alkaline stress increased the NH_4_^+^-nitrogen content in the leaves of tomato seedlings, whereas application of exogenous spermidine can decrease the NH_4_^+^-nitrogen content induced by saline–alkaline stress. Inconsistent with these findings, our results also indicate that rice plants accumulated an excessive quantity of NH_4_^+^ in their shoots in response to saline–alkaline stress, whereas the application of magnetized water mitigated the saline–alkaline stress-induced elevation of NH_4_^+^ (Figure 3E). Prior studies also shown that when plants were subjected to higher levels of saline–alkaline stress, the rhizosphere did not contain much NH_4_^+^ because almost all of the NH_4_^+^ was transformed into NH_3_ [16], which prevented the plants from absorbing additional NH_4_^+^. In our study, it was found that saline–alkaline stress lowered the NH_4_^+^-nitrogen level in the roots of rice plants, whereas the application of magnetized water mitigated this reduction (Figure 3F). These findings indicated that magnetized water treatment may improve rice plants’ tolerance to saline–alkaline stress by reducing the total amount of NH_4_^+^-nitrogen concentration in their roots. To further investigate the molecular mechanisms underlying the change in NH_4_^+^-nitrogen content in Nipponbare plants caused by the application of magnetized water, the expression patterns of genes involved in NH_4_^+^ absorption were studied. Generally, NH_4_^+^ uptake was mediated by two distinct systems: the high-affinity uptake system and the low-affinity uptake system. [39]. For the low-affinity system, the proteins responsible for NH_4_^+^ uptake have not yet been discovered, however, it has been proposed that the low-affinity system might be mediated by aquaporins and cation channels [40]. While for the high-affinity system, it was discovered that the *OsAMTs* gene families are responsible for NH_4_^+^ uptake [30]. According to several investigations, the *OsAMTs* gene expression, including *OsAMT1;2*, *OsAMT2;1*, *OsAMT2;2*, *OsAMT2;3*, *OsAMT3;1*, *OsAMT3;2*, and *OsAMT3;3*, was up-regulated under saline–alkaline stress conditions as an adaptive response to the reduction of NH_4_^+^ in roots [16]. For example, Wang et al. (2012), discovered that the NH_4_^+^ transporter genes family (*OsAM*T) was increased in rice roots to adapt to NH_4_^+^ deficiency induced by saline–alkaline stress [41]. Lin et al. (2020) conducted a transcriptome study of rice root’s status under saline–alkaline stress and found that the expression levels of *OsAMT2;1* and *OsAMT2;3* were up-regulated under high alkaline stress conditions [42]. Consistent with these results, the expression levels of *OsAMT1;2*, *OsAMT2;1*, *OsAMT2;2*, *OsAMT3;1*, *OsAMT3;2*, and *OsAMT3;3* in rice roots in our study were significantly up-regulated under a saline–alkaline stress environment, and the degree of the up-regulation was greater in plants when magnetized water was applied (Figure 6, Figure 7 and Figure 8). These findings may suggest that rice seedlings have increased the expression of genes involved in NH_4_^+^ absorption to compensate for a decline in NH_4_^+^-nitrogen concentration in their roots. In general, saline–alkaline stress modifies the expression levels of *OsAMT1;2*, *OsAMT2;1*, *OsAMT2;2*, *OsAMT3;1*, *OsAMT3;2*, and *OsAMT3;3* in plant shoots [37]. Inconsistent with these findings, our research also reveals that exposure to saline–alkaline stress modifies the expression of genes involved in NH_4_^+^ absorption in the shoots of rice plants. This modification may be the result of decreasing nitrogen levels in plants, which compensate for a decrease in NH_4_^+^-nitrogen content in shoots.

Under saline–alkaline stress, the characteristics of the root system have a crucial influence on the nitrogen acquisition of plants [18]. The observation that seedlings irrigated with magnetized water had a higher nitrogen content than those irrigated with non-magnetized water under saline–alkaline stress conditions prompted us to investigate if their root systems varied in response to magnetized water treatment. Under saline–alkaline circumstances, the adventitious root number, total root length, and root surface area of seedlings exposed to magnetized water were considerably greater than those of seedlings not exposed to magnetized water (Figure 8). These findings imply that treatment with magnetized water can contribute to the development of a more extensive root system in Nipponbare seedlings, which may promote nitrogen acquisition, thereby enhancing tolerance to nitrogen deficiency caused by saline–alkaline stress. In accordance with our findings, other studies have also demonstrated that irrigation with magnetized water can stimulate root growth, hence mitigating the growth-inhibiting effects of stress on plants such as cotton [32], Populus [43], wheat, and rice [44].

## 4. Materials and Methods

### 4.1. Plant Materials and Germination Treatments

In this experiment, *Oryza sativa* L. ssp. *Japonica cv.* Nipponbare was employed. The seeds were germinated in tap water at 37 °C for two days before being moved to moist tissue paper at 30 °C in the dark for two days. The seedlings were then transferred to a nutritional solution containing (mM): 1.425 NH_4_NO_3_, 0.42 NaH_2_PO_4_, 0.510 K_2_SO_4_, 0.998 CaCl_2_, 1.643 MgSO_4_, 0.168 Na_2_SiO_3_, 0.125 Fe-EDTA, 0.019 H_3_BO_3_, 0.009 MnCl_2_, 0.155 CuSO_4_, 0.152 ZnSO_4_, and 0.075 Na_2_MoO_4_, cultivated in a growth chamber that was kept at a constant 30 °C/22 °C (day/night) temperature with a 14-hour photoperiod and a relative humidity of about 70%. Half of the seedlings were transferred to soil with 60 mM NaHCO_3_ and a pH of 8.5 after spending 3 weeks growing in the solution. The remainder of the plants were left in the original soil, which was just ordinary soil. In order to assess the effects of magnetized water on the growth of Nipponbare seedlings under saline–alkaline stress, half of these seedlings were irrigated with magnetized water for two weeks, while the other half received tap water as a control.

### 4.2. Measurements of Plant Growth

The shoots and roots of the rice seedlings were collected and oven-dried at 75 °C for 2 days until their weight reached a steady level. Roots were scanned with an Epson digital scanner (Expression 10000XL, Epson (China) Co., Ltd, Beijing, China) and processed with the WinRHIZO/WinFOLIA program in order to investigate root morphological parameters (Regent Instruments Inc., Quebec, Canadian).

### 4.3. Measurements of Chlorophyll (CHL) Concentration

Chlorophyll concentration was determined following the procedures outlined by Li et al. (2016). In brief, to determine CHL concentration in rice plants, newly produced leaves were plucked, weighed, and extracted with aqueous ethanol (95% *v/v*). Absorbance (A) readings of the supernatant were recorded at wavelengths of 663 and 645 nm. The total CHL content was calculated as 8.02A663 + 20.21A645 and expressed as mg chlorophyll per gram of fresh mass.

### 4.4. Measurements of Photosynthetic Characteristics

Photosynthetic rates of rice seedlings were recorded between 8:30 and 11:30 with an LI-6400 XT portable photosynthesis system equipped with an LED leaf cuvette (Li-Cor, 146 Lincoln, NE, USA). The leaves in the chamber were artificially illuminated using a red–blue 6400-02B LED light source that was mounted to the sensor head and provided with continuous illumination (1000 mol m^−2^ s^−1^ photosynthetic photon flux density) and an ambient CO_2_ concentration of approximately 500 mol CO_2_ mol. At least 15 individual rice plants were chosen for evaluation of photosynthetic rates for each stress treatment.

### 4.5. Determination of Total Nitrogen Concentration, NO_3_^−^-Nitrogen Concentration, and NH_4_^+^-Nitrogen Concentration in Rice Plants

Total soil nitrogen was measured using the semi-micro Kjeldahl method with a small modification, as described by Li et al. (2013) [45].

The NO_3_^−^-nitrogen concentration was measured using the method described by Luo et al. (2013) [46]. Fine powder (~100 mg) was extracted for 1 h in 1 mL deionized water at 45 °C. After centrifugation (5000× *g*, 20 °C, 15 min), 0.2 mL of the supernatant was combined with 0.8 mL of 5% (*w/v*) salicylic acid (SA) in H_2_SO_4_ concentration. After 20 min of room-temperature incubation, 19 mL of 2 M NaOH was added to increase the pH to greater than 12. After being cooled to room temperature, the absorbance of the solution was determined spectrophotometrically at 410 nm.

The NH_4_^+^ concentration in the shoots and roots was determined based on the methods described by Luo et al. (2013) [46]. In brief, ~100 mg of fine powder was extracted in an extraction solution (1 mL 100 mM HCl and 500 µL chloroform), which was then shaken for 15 min at 4 °C. After that, the solution was centrifuged (10,000× *g*, 4 °C, 10 min) and the aqueous phase was transferred to a fresh tube and mixed with 50 mg of activated charcoal before being centrifuged again (12,000× *g*, 4 °C, 5 min). Then, 100 µL of the extraction solution was blended with 500 µL 1% (*w/v*) phenol–0.005% (*w/v*) sodium nitroprusside solution. Following this, 500 µL 1% (*v/v*) sodium hypochlorite–0.5% (*w/v*) sodium hydroxide solution was added. The mixture was incubated at 37 °C for 30 min and spectrophotometrically measured at 620 nm.

### 4.6. Determination of Total Nitrogen Content, Available Nitrogen Content, NO_3_^−^-Nitrogen Content, and NH_4_^+^-Nitrogen Content in the Soil

The rhizospheric soil from both loose soil and cohesive soil from the rice plant roots was collected, mixed, naturally dried, powdered, sieved, and divided into two parts. One part of fresh soil was naturally air dried, ground using the quarter method, and then passed through a 100 mesh sieve to determine soil total nitrogen. The other soil sample was stored in a refrigerator at 4 °C to determine soil NO_3_^−^-nitrogen and NH_4_^+^-nitrogen. The soil’s total nitrogen was measured using the semi-micro Kjeldahl method and a Kjeltec System 1026 Distilling Unit in accordance with the method described by Ivančič and Degobbis (1984) [47]. Soil’s NH_4_^+^-nitrogen and NO_3_^−^-nitrogen contents were measured using a FIAstar 5000 Analyzer FOSS TECATOR instrument according to the suggested method by Patterson et al. (2010) [48]. The soil’s available nitrogen is the sum of soil NO_3_^−^- nitrogen and NH_4_^+^-nitrogen.

### 4.7. RNA Isolation and Real-Time RT-PCR

The procedures were detailed in Li et al. (2016) [6]. In brief, total RNA was extracted with Trizol (Invitrogen, Carlsbad, CA, USA) reagent and treated with RNase-free DNase I. (Promega, Madison, WI, USA). Total RNAs were reverse transcribed into first-strand cDNA in a 20-L volume with M-MLV reverse transcriptase (Promega). With an Applied Biosystems Step one TM Real-Time PCR apparatus, real-time PCR was performed on an optical 96-well plate. Each reaction contained 5 L of diluted cDNA, 12.5 L of SYBR GreenER qPCR SuperMix Universal (Invitrogen), 0.5 L of Rox Reference Dye, 1 L each of 10 M forward and reverse primers, and 5 L of sterile water. The heat cycle was as follows: 95 °C for 10 min, followed by 40 cycles of 95 °C for 30 s, 60 °C for 30 s, and 72 °C for 30 s. The primers employed for each gene are provided in Table S1. Internal control was implemented using Actin (GenBank accession number AB047313). The relative level of expression was evaluated using the comparative Ct technique.

### 4.8. Statistical Analysis

For the analysis of variance, the SAS statistical software was utilized. Significant differences between treatments were analyzed using a Student’s *t*-test.

## 5. Conclusions

We present experimental evidence supporting the modulation of rice seedling responses to saline–alkaline stress by irrigation with magnetized water. Specifically, we demonstrate that rice seedlings treated with magnetic water are more resilient to saline–alkaline stress. Our findings revealed that a highly efficient nitrogen acquisition enhanced by magnetized water may give better tolerance to saline–alkaline stress in rice plants. These findings provide crucial information for our mechanistic understanding of rice plants in response to saline–alkaline stress.

## Supplementary Materials

The supporting information can be downloaded at: https://www.mdpi.com/article/10.3390/ijms231710048/s1.
