# Supplementary material for: Irrigation with Magnetized Water Alleviates the Harmful Effect of Saline–Alkaline Stress on Rice Seedlings
Source: Int J Mol Sci. 2022 Sep 2;23(17):10048. doi: 10.3390/ijms231710048 (PMC9456538; doi:10.3390/ijms231710048)
Supplement: Supplementary file 1 [file ijms-23-10048-s001.zip › ijms-1878836-supplementary.pdf]

Table S1. Primers used for quantitative real-time PCR in this study

**Gene**	**Forward primer 5' → 3'**	**Reverse primer 5' → 3'**
OsNRT1;1	GGGCAGAGTTCAGCAATCG	GGAAGGACGCCGCAGGT
OsNRT1;2	GCGGCGAGTCCCTGAG	CGACGGCGTAGATGAATGA
OsNRT2;1	ACGGCACAAAGTACAAGACG	CCACTGCGGGAAGTAGATG
OsAMT1;1	GCCTCCAACAGCAACAACC	CCAAACAGAAACTGGCAATCA
OsAMT1;2	CACGGTGGCGATGAAAGG	TTGGAGATGGTGGTGAAGGAC
OsAMT1;3	TCAAGCAGGTCCCACAGG	TGAGGAAGGCGGAGTAGATG
OsAMT2;1	GATGAATCACGCCGAAACAC	GCACGGACGAATCGCTACTT
OsAMT2;2	CGACCAAGGACAGGGAGA	CACGGCGAGCGAGGAG
OsAMT2;3	GTTCACCCCGCTCTGGC	CCGCTCCCTGTCGCTCTT
OsAMT3;1	CCAACTGCTGAAAAGTGAAAACG	TGCTTCGCATACGGCTGAC
OsAMT3;2	CCCAGTTCGGCAAGCAG	TGGCGAGGCAGATGAGG
OsAMT3;3	GAGATTCCCGCCCAACAA	TCCACCCAAGCCACAGC
Actin	ACCACAGGTATTGTGTTGGACTC	AGAGCATATCCTTCATAGATGGG
